# Supplementary material for: Electrophysiological correlates of focused attention on low- and high-distressed tinnitus
Source: PLoS One. 2020 Aug 5;15(8):e0236521. doi: 10.1371/journal.pone.0236521 (PMC7406215; doi:10.1371/journal.pone.0236521)
Supplement: S2 Table — Items in bold are significant based on p-values. (PDF) [file pone.0236521.s002.pdf]

S2 Table. Post-hoc pairwise t-tests results for the contrast: body focus condition (BFC) versus tinnitus focus condition (TFC) calculated in each cluster for the whole study sample (n=67). Items in bold are significant based on p-values.

		M_{Diff}	CI L	CI U	p-value	FDR-adjusted p -value			M_{Diff}	CI L	CI U	p-value	FDR-adjusted p -value
Delta	LA	-0.029	-0.053	-0.006	**0.015 ****	0.409	Low Beta	LA	0.003	-0.019	0.026	0.795	0.980
	RA	-0.019	-0.042	0.004	0.106	0.774		RA	0.002	-0.019	0.024	0.856	0.981
	LM	-0.011	-0.035	0.012	0.338	0.860		LM	0.000	-0.024	0.024	0.979	0.985
	RM	-0.005	-0.026	0.017	0.654	0.893		RM	0.008	-0.013	0.029	0.473	0.893
	CE	0.005	-0.012	0.022	0.571	0.893		CE	0.005	-0.021	0.031	0.734	0.979
	LP	-0.005	-0.026	0.016	0.633	0.893		LP	0.016	-0.011	0.046	0.241	0.819
	RP	0.000	-0.021	0.023	0.985	0.985		RP	0.011	-0.019	0.043	0.509	0.893
Theta	LA	0.001	-0.018	0.020	0.937	0.981	Middle Beta	LA	0.012	-0.015	0.041	0.397	0.893
	RA	0.003	-0.015	0.022	0.773	0.980		RA	-0.001	-0.023	0.020	0.905	0.981
	LM	0.007	-0.013	0.028	0.500	0.893		LM	0.016	-0.009	0.041	0.217	0.819
	RM	0.003	-0.017	0.023	0.800	0.980		RM	0.005	-0.017	0.028	0.644	0.893
	CE	0.007	-0.013	0.029	0.517	0.893		CE	0.006	-0.014	0.025	0.579	0.893
	LP	0.003	-0.020	0.025	0.830	0.981		LP	0.006	-0.014	0.026	0.611	0.893
	RP	0.001	-0.021	0.023	0.934	0.981		RP	0.007	-0.016	0.031	0.539	0.893
Low Alpha	LA	0.019	-0.014	0.052	0.268	0.819	High Beta	LA	0.027	-0.006	0.064	0.108	0.774
	RA	0.018	-0.015	0.049	0.298	0.834		RA	-0.006	-0.031	0.018	0.643	0.893
	LM	0.019	-0.015	0.052	0.267	0.819		LM	0.021	-0.010	0.054	0.194	0.819
	RM	0.017	-0.014	0.048	0.278	0.819		RM	0.002	-0.025	0.029	0.890	0.981
	CE	0.015	-0.022	0.052	0.441	0.893		CE	0.013	-0.006	0.033	0.196	0.819
	LP	0.020	-0.024	0.063	0.385	0.893		LP	0.018	-0.005	0.045	0.133	0.774
	RP	0.013	-0.029	0.055	0.564	0.893		RP	0.015	-0.015	0.043	0.320	0.853
High Alpha	LA	0.028	-0.011	0.069	0.173	0.819	Gamma	LA	-0.002	-0.051	0.048	0.946	0.981
	RA	0.031	-0.006	0.072	0.107	0.774		RA	-0.046	-0.083	-0.011	**0.012 ****	0.409
	LM	0.024	-0.015	0.065	0.234	0.819		LM	0.020	-0.038	0.079	0.491	0.893
	RM	0.028	-0.008	0.067	0.138	0.774		RM	0.013	-0.036	0.062	0.619	0.893
	CE	0.035	-0.010	0.081	0.127	0.774		CE	0.011	-0.023	0.052	0.596	0.893
	LP	0.045	-0.003	0.096	0.066	0.774		LP	-0.001	-0.040	0.039	0.936	0.981
	RP	0.043	-0.008	0.095	0.100	0.774		RP	0.005	-0.040	0.049	0.805	0.980

MDiff – factor score mean difference; CI – 95% confidential interval; L – lower bound; U – upper bound; LA – left anterior; RA – right anterior; LM – left middle; RM – right middle; CE – central; LP – left posterior; RP – right posterior; LA – left anterior; RA – right anterior.
