# Supplementary material for: Electrophysiological correlates of focused attention on low- and high-distressed tinnitus
Source: PLoS One. 2020 Aug 5;15(8):e0236521. doi: 10.1371/journal.pone.0236521 (PMC7406215; doi:10.1371/journal.pone.0236521)
Supplement: S3 Table — (PDF) [file pone.0236521.s003.pdf]

S3 Table. The mean absolute power and standard error (in brackets) values for different frequency bands in the contrast: body focus condition (BFC) versus tinnitus focus condition (TFC), calculated in each cluster separately for the low tinnitus-related distress (LD) and high tinnitus-related distress (HD) group.

		LD		HD				LD		HD	
		TFC	BFC	TFC	BFC			TFC	BFC	TFC	BFC
Delta	LA	**0.976 (0.031)**	**0.954 (0.026)**	0.968 (0.030)	0.975 (0.025)	Low Beta	LA	0.305 (0.040)	0.312 (0.041)	0.401 (0.039)	0.412 (0.040)
	RA	0.998 (0.030)	0.971 (0.029)	0.970 (0.029)	0.958 (0.028)		RA	0.322 (0.042)	0.320 (0.043)	0.402 (0.041)	0.408 (0.042)
	LM	0.875 (0.025)	0.854 (0.025)	0.911 (0.024)	0.910 (0.024)		LM	0.392 (0.044)	0.395 (0.046)	0.539 (0.044	0.538 (0.045)
	RM	0.895 (0.027)	0.897 (0.027)	0.913 (0.026)	0.900 (0.027)		RM	0.405 (0.047)	0.401 (0.047)	0.506 (0.046)	0.526 (0.046)
	CE	1.035 (0.025)	1.039 (0.025)	1.065 (0.025)	1.071 (0.025)		CE	0.537 (0.047)	0.526 (0.049)	0.634 (0.046)	0.654 (0.048)
	LP	0.894 (0.028)	0.893 (0.028)	0.957 (0.028)	0.948 (0.028)		LP	0.541 (0.054)	0.549 (0.056)	0.683 (0.053)	0.707 (0.055)
	RP	0.889 (0.030)	0.905 (0.031)	0.936 (0.030)	0.921 (0.030)		RP	0.563 (0.057)	0.563 (0.058)	0.672 (0.056)	0.692 (0.057)
Theta	LA	0.761 (0.692)	0.763 (0.033)	0.793 (0.034)	0.809 (0.032)	Middle Beta	LA	0.204 (0.039)	0.209 (0.040)	0.288 (0.039)	0.323 (0.039)
	RA	0.788 (0.718)	0.782 (0.034)	0.806 (0.035)	0.819 (0.033)		RA	0.211 (0.040)	0.211 (0.041)	0.298 (0.040)	0.295 (0.041)
	LM	0.706 (0.640)	0.709 (0.033)	0.781 (0.033)	0.792 (0.032)		LM	0.303 (0.046)	0.308 (0.046)	0.395 (0.046)	0.421 (0.045)
	RM	0.741 (0.669)	0.743 (0.034)	0.778 (0.035)	0.781 (0.034)		RM	0.300 (0.049)	0.301 (0.048)	0.389 (0.048)	0.400 (0.047)
	CE	0.936 (0.866)	0.939 (0.034)	0.988 (0.034)	0.999 (0.034)		CE	0.377 (0.046)	0.371 (0.047)	0.484 (0.045)	0.501 (0.046)
	LP	0.758 (0.684)	0.755 (0.038)	0.842 (0.037)	0.850 (0.037)		LP	0.326 (0.047)	0.329 (0.048)	0.476 (0.047)	0.484 (0.047)
	RP	0.750 (0.670)	0.750 (0.040)	0.811 (0.039)	0.813 (0.039)		RP	0.329 (0.048)	0.333 (0.049)	0.445 (0.047)	0.456 (0.049)
Low Alpha	LA	0.614 (0.069)	0.619 (0.066)	**0.686 (0.068)**	**0.734 (0.065)**	High Beta	LA	0.457 (0.037)	0.470 (0.041)	0.505 (0.037)	0.547 (0.040)
	RA	0.660 (0.071)	0.652 (0.069)	**0.709 (0.070)**	**0.752 (0.068)**		RA	0.462 (0.037)	0.455 (0.038)	0.505 (0.036)	0.501 (0.037)
	LM	0.593 (0.072)	0.601 (0.071)	0.699 (0.070)	0.729 (0.070)		LM	0.512 (0.044)	0.554 (0.043)	0.603 (0.043)	0.604 (0.043)
	RM	0.634 (0.074)	0.640 (0.073)	0.707 (0.073)	0.735 (0.072)		RM	0.535 (0.042)	0.518 (0.040)	0.554 (0.041)	0.575 (0.039)
	CE	0.844 (0.074)	0.835 (0.074)	0.918 (0.073)	0.957 (0.073)		CE	0.572 (0.036)	0.576 (0.039)	0.668 (0.035)	0.691 (0.038)
	LP	0.747 (0.081)	0.744 (0.079)	0.899 (0.080)	0.941 (0.077)		LP	0.500 (0.038)	0.528 (0.040	0.647 (0.037)	0.656 (0.039)
	RP	0.774 (0.086)	0.767 (0.083)	0.877 (0.085)	0.909 (0.082)		RP	0.525 (0.040)	0.525 (0.041)	0.607 (0.040)	0.636 (0.041)
High Alpha	LA	0.489 (0.058)	0.513 (0.062)	0.558 (0.057)	0.599 (0.061)	Gamma	LA	0.296 (0.051)	0.310 (0.056)	0.272 (0.050)	0.254 (0.055)
	RA	0.512 (0.061)	0.533 (0.065)	**0.570 (0.060)**	**0.613 (0.064)**		RA	0.293 (0.049)	0.248 (0.047)	**0.245 (0.048)**	**0.198 (0.047)**
	LM	0.539 (0.062)	0.559 (0.065)	0.658 (0.061)	0.686 (0.064)		LM	0.312 (0.064)	0.363 (0.059)	0.317 (0.063)	0.307 (0.058)
	RM	0.553 (0.066)	0.568 (0.069)	0.644 (0.065)	0.684 (0.068)		RM	0.309 (0.056)	0.304 (0.051)	0.256 (0.055)	0.286 (0.051)
	CE	0.766 (0.070)	0.772 (0.075)	**0.872 (0.069)**	**0.935 (0.074)**		CE	0.274 (0.047)	0.270 (0.045)	0.232 (0.046)	0.258 (0.044)
	LP	0.832 (0.083)	0.853 (0.087)	**1.001 (0.082)**	**1.070 (0.086)**		LP	0.204 (0.038)	0.213 (0.036)	0.225 (0.037)	0.214 (0.036)
	RP	0.882 (0.087)	0.888 (0.092)	**0.993 (0.086)**	**1.073 (0.091)**		RP	0.240 (0.044)	0.225 (0.040)	0.189 (0.044)	0.214 (0.039)

LA – left anterior; RA – right anterior; LM – left middle; RM – right middle; CE – central; LP – left posterior; RP – right posterior. Items in bold are significant based on p-values.
