# Supplementary material for: Electrophysiological correlates of focused attention on low- and high-distressed tinnitus
Source: PLoS One. 2020 Aug 5;15(8):e0236521. doi: 10.1371/journal.pone.0236521 (PMC7406215; doi:10.1371/journal.pone.0236521)
Supplement: S4 Table — Items in bold are significant based on p-values. (PDF) [file pone.0236521.s004.pdf]

S4 Table. Post-hoc pairwise t-tests results for the contrast: body focus condition (BFC) versus tinnitus focus condition (TFC), calculated in each cluster separately for the high tinnitus-related distress (HD) and low tinnitus-related distress (LD) group. Items in bold are significant based on p-values.

HD

		M_{Diff}	CI L	CI U	p-value	FDR-adjusted p -value
Delta	LA	-0.022	-0.053	0.012	0.186	0.800
	RA	-0.012	-0.043	0.020	0.412	0.993
	LM	-0.002	-0.036	0.033	0.903	0.993
	RM	-0.012	-0.041	0.014	0.386	0.993
	CE	0.006	-0.018	0.031	0.653	0.993
	LP	-0.009	-0.036	0.021	0.503	0.993
	RP	-0.015	-0.043	0.013	0.270	0.958
Theta	LA	0.009	-0.018	0.034	0.466	0.993
	RA	0.013	-0.013	0.038	0.306	0.993
	LM	0.012	-0.016	0.038	0.410	0.993
	RM	0.003	-0.026	0.031	0.857	0.993
	CE	0.011	-0.017	0.036	0.414	0.993
	LP	0.008	-0.022	0.037	0.639	0.993
	RP	0.002	-0.031	0.032	0.927	0.993
Low Alpha	LA	0.039	0.002	0.075	**0.034***	0.543
	RA	0.043	0.004	0.082	**0.028***	0.543
	LM	0.030	-0.014	0.073	0.178	0.797
	RM	0.029	-0.011	0.066	0.164	0.765
	CE	0.039	-0.007	0.085	0.088	0.765
	LP	0.042	-0.013	0.101	0.152	0.765
	RP	0.032	-0.023	0.087	0.274	0.958
High Alpha	LA	0.035	-0.006	0.074	0.098	0.765
	RA	0.042	0.001	0.081	**0.050***	0.699
	LM	0.028	-0.021	0.073	0.262	0.958
	RM	0.040	-0.006	0.082	0.082	0.765
	CE	0.063	0.006	0.116	**0.026***	0.543
	LP	0.068	0.017	0.119	**0.006****	0.336
	RP	0.080	0.025	0.133	**0.004****	0.336
Low Beta	LA	0.000	-0.030	0.028	0.989	0.993
	RA	0.006	-0.020	0.032	0.713	0.993
	LM	-0.002	-0.041	0.033	0.899	0.993
	RM	0.020	-0.007	0.046	0.130	0.765
	CE	0.020	-0.014	0.052	0.224	0.864
	LP	0.025	-0.008	0.057	0.150	0.765
	RP	0.020	-0.018	0.061	0.314	0.993
Middle Beta	LA	0.015	-0.021	0.063	0.527	0.993
	RA	-0.003	-0.026	0.021	0.781	0.993
	LM	0.026	-0.009	0.063	0.148	0.765
	RM	0.010	-0.016	0.037	0.442	0.993
	CE	0.018	-0.007	0.045	0.212	0.847
	LP	0.008	-0.018	0.035	0.571	0.993
	RP	0.011	-0.019	0.037	0.519	0.993
High Beta	LA	0.041	-0.009	0.108	0.122	0.765
	RA	-0.005	-0.035	0.023	0.789	0.993
	LM	0.001	-0.039	0.040	0.973	0.993
	RM	0.021	-0.004	0.044	0.098	0.765
	CE	0.022	-0.003	0.050	0.070	0.765
	LP	0.009	-0.016	0.032	0.494	0.993
	RP	0.029	-0.004	0.058	0.078	0.765
Gamma	LA	-0.018	-0.083	0.059	0.581	0.993
	RA	-0.048	-0.098	-0.008	**0.020***	0.543
	LM	-0.010	-0.077	0.051	0.725	0.993
	RM	0.030	-0.028	0.087	0.320	0.993
	CE	0.026	-0.029	0.103	0.464	0.993
	LP	-0.011	-0.056	0.033	0.659	0.993
	RP	0.025	-0.030	0.080	0.376	0.993

M_{Diff} – factor score mean difference; CI – 95% confidential interval; L – lower bound; U – upper bound; LA – left anterior; RA – right anterior; LM – left middle; RM – right middle; CE – central; LP – left posterior; RP – right posterior; LA – left anterior; RA – right anterior; * $p < 0.05$; ** $p < 0.01$.

S4 Table continuation

LD													
		M $_{Diff}$	CI		p-value	FDR-adjusted p -value			M $_{Diff}$	CI		p-value	FDR-adjusted p -value
			L	U						L	U		
Delta	LA	-0.037	-0.072	-0.003	**0.032***	0.543	Low Beta	LA	0.007	-0.028	0.042	0.715	0.993
	RA	-0.027	-0.060	0.005	0.104	0.765		RA	-0.002	-0.033	0.033	0.917	0.993
	LM	-0.021	-0.057	0.009	0.198	0.821		LM	0.002	-0.030	0.037	0.903	0.993
	RM	0.002	-0.032	0.039	0.881	0.993		RM	-0.005	-0.036	0.029	0.787	0.993
	CE	0.004	-0.020	0.029	0.739	0.993		CE	-0.011	-0.051	0.028	0.575	0.993
	LP	-0.001	-0.031	0.030	0.915	0.993		LP	0.008	-0.034	0.057	0.725	0.993
	RP	0.015	-0.017	0.052	0.364	0.993		RP	0.001	-0.046	0.051	0.993	0.993
Theta	LA	-0.007	-0.037	0.023	0.559	0.993	Middle Beta	LA	0.009	-0.030	0.050	0.689	0.993
	RA	-0.007	-0.034	0.024	0.573	0.993		RA	0.000	-0.035	0.037	0.949	0.993
	LM	0.002	-0.027	0.033	0.893	0.993		LM	0.005	-0.032	0.043	0.781	0.993
	RM	0.003	-0.027	0.034	0.853	0.993		RM	0.000	-0.035	0.036	0.967	0.993
	CE	0.003	-0.028	0.037	0.893	0.993		CE	-0.006	-0.037	0.023	0.639	0.993
	LP	-0.003	-0.039	0.033	0.891	0.993		LP	0.003	-0.026	0.037	0.869	0.993
	RP	0.000	-0.034	0.034	0.985	0.993		RP	0.004	-0.032	0.042	0.785	0.993
Low Alpha	LA	-0.001	-0.062	0.056	0.983	0.993	High Beta	LA	0.013	-0.027	0.051	0.500	0.993
	RA	-0.008	-0.063	0.043	0.773	0.993		RA	-0.008	-0.042	0.033	0.659	0.993
	LM	0.008	-0.048	0.059	0.707	0.993		LM	0.042	-0.009	0.091	0.128	0.765
	RM	0.005	-0.043	0.053	0.775	0.993		RM	-0.017	-0.063	0.034	0.480	0.993
	CE	-0.009	-0.070	0.051	0.757	0.993		CE	0.003	-0.028	0.033	0.827	0.993
	LP	-0.003	-0.072	0.059	0.949	0.993		LP	0.028	-0.011	0.074	0.158	0.765
	RP	-0.007	-0.070	0.062	0.853	0.993		RP	0.000	-0.049	0.049	0.945	0.993
High Alpha	LA	0.020	-0.048	0.093	0.601	0.993	Gamma	LA	-0.018	-0.054	0.082	0.691	0.993
	RA	0.020	-0.043	0.087	0.559	0.993		RA	-0.048	-0.100	0.011	0.130	0.765
	LM	0.020	-0.042	0.086	0.541	0.993		LM	-0.010	-0.048	0.145	0.310	0.993
	RM	0.016	-0.044	0.077	0.577	0.993		RM	0.030	-0.078	0.072	0.927	0.993
	CE	0.007	-0.064	0.080	0.845	0.993		CE	0.026	-0.044	0.035	0.823	0.993
	LP	0.021	-0.061	0.106	0.617	0.993		LP	-0.011	-0.054	0.073	0.801	0.993
	RP	0.006	-0.079	0.094	0.879	0.993		RP	0.025	-0.086	0.053	0.681	0.993

M_{Diff} – factor score mean difference; CI – 95% confidential interval; L – lower bound; U – upper bound; LA – left anterior; RA – right anterior; LM – left middle; RM – right middle; CE – central; LP – left posterior; RP – right posterior; LA – left anterior; RA – right anterior; * p < 0.05; ** p < 0.01.
