# Supplementary material for: Electrophysiological correlates of focused attention on low- and high-distressed tinnitus
Source: PLoS One. 2020 Aug 5;15(8):e0236521. doi: 10.1371/journal.pone.0236521 (PMC7406215; doi:10.1371/journal.pone.0236521)
Supplement: S5 Table — (PDF) [file pone.0236521.s005.pdf]

S5 Table. The mean absolute power and standard error (in brackets) values for different frequency bands in the contrast: high tinnitus-related distress (HD) versus low tinnitus-related distress (LD) group, calculated in each cluster separately for the body focus condition (BFC) and tinnitus focus condition (TFC).

		BFC		TFC				BFC		TFC	
		HD	LD	HD	LD			HD	LD	HD	LD
Delta	LA	0.975 (0.025)	0.954 (0.026)	0.968 (0.030)	0.976 (0.031)	Low Beta	LA	0.412 (0.040)	0.312 (0.041)	0.401 (0.039)	0.305 (0.040)
	RA	0.958 (0.028)	0.971 (0.029)	0.970 (0.029)	0.998 (0.030)		RA	0.408 (0.042)	0.320 (0.043)	0.402 (0.041)	0.322 (0.042)
	LM	0.910 (0.024)	0.854 (0.025)	0.911 (0.024)	0.875 (0.025)		LM	**0.538 (0.045)**	**0.395 (0.046)**	**0.539 (0.044)**	**0.392 (0.044)**
	RM	0.900 (0.027)	0.897 (0.027)	0.913 (0.026)	0.895 (0.027)		RM	0.526 (0.046)	0.401 (0.047)	0.506 (0.046)	0.405 (0.047)
	CE	1.071 (0.025)	1.039 (0.025)	1.065 (0.025)	1.035 (0.025)		CE	0.654 (0.048)	0.526 (0.049)	0.634 (0.046)	0.537 (0.047)
	LP	0.948 (0.028)	0.893 (0.028)	0.957 (0.028)	0.894 (0.028)		LP	**0.707 (0.055)**	**0.549 (0.056)**	0.683 (0.053)	0.541 (0.054)
	RP	0.921 (0.030)	0.905 (0.031)	0.936 (0.030)	0.889 (0.030)		RP	0.692 (0.057)	0.563 (0.058)	0.672 (0.056)	0.563 (0.057)
Theta	LA	0.809 (0.032)	0.763 (0.033)	0.793 (0.034)	0.761 (0.692)	Middle Beta	LA	0.323 (0.039)	0.209 (0.040)	0.288 (0.039)	0.204 (0.039)
	RA	0.819 (0.033)	0.782 (0.034)	0.806 (0.035)	0.788 (0.718)		RA	0.295 (0.041)	0.211 (0.041)	0.298 (0.040)	0.211 (0.040)
	LM	0.792 (0.032)	0.709 (0.033)	0.781 (0.033)	0.706 (0.640)		LM	0.421 (0.045)	0.308 (0.046)	0.395 (0.046)	0.303 (0.046)
	RM	0.781 (0.034)	0.743 (0.034)	0.778 (0.035)	0.741 (0.669)		RM	0.400 (0.047)	0.301 (0.048)	0.389 (0.048)	0.300 (0.049)
	CE	0.999 (0.034)	0.939 (0.034)	0.988 (0.034)	0.936 (0.866)		CE	**0.501 (0.046)**	**0.371 (0.047)**	0.484 (0.045)	0.377 (0.046)
	LP	0.850 (0.037)	0.755 (0.038)	0.842 (0.037)	0.758 (0.684)		LP	**0.484 (0.047)**	**0.329 (0.048)**	**0.476 (0.047)**	**0.326 (0.047)**
	RP	0.813 (0.039)	0.750 (0.040)	0.811 (0.039)	0.750 (0.670)		RP	0.456 (0.049)	0.333 (0.049)	0.445 (0.047)	0.329 (0.048)
Low Alpha	LA	0.734 (0.065)	0.619 (0.066)	0.686 (0.068)	0.614 (0.069)	High Beta	LA	0.547 (0.040)	0.470 (0.041)	0.505 (0.037)	0.457 (0.037)
	RA	0.752 (0.068)	0.652 (0.069)	0.709 (0.070)	0.660 (0.071)		RA	0.501 (0.037)	0.455 (0.038)	0.505 (0.036)	0.462 (0.037)
	LM	0.729 (0.070)	0.601 (0.071)	0.699 (0.070)	0.593 (0.072)		LM	0.604 (0.043)	0.554 (0.043)	0.603 (0.043)	0.512 (0.044)
	RM	0.735 (0.072)	0.640 (0.073)	0.707 (0.073)	0.634 (0.074)		RM	0.575 (0.039)	0.518 (0.040)	0.554 (0.041)	0.535 (0.042)
	CE	0.957 (0.073)	0.835 (0.074)	0.918 (0.073)	0.844 (0.074)		CE	**0.691 (0.038)**	**0.576 (0.039)**	0.668 (0.035)	0.572 (0.036)
	LP	0.941 (0.077)	0.744 (0.079)	0.899 (0.080)	0.747 (0.081)		LP	**0.656 (0.039)**	**0.528 (0.040)**	**0.647 (0.037)**	**0.500 (0.038)**
	RP	0.909 (0.082)	0.767 (0.083)	0.877 (0.085)	0.774 (0.086)		RP	0.636 (0.041)	0.525 (0.041)	0.607 (0.040)	0.525 (0.040)
High Alpha	LA	0.599 (0.061)	0.513 (0.062)	0.558 (0.057)	0.489 (0.058)	Gamma	LA	0.254 (0.055)	0.310 (0.056)	0.272 (0.050)	0.296 (0.051)
	RA	0.613 (0.064)	0.533 (0.065)	0.570 (0.060)	0.512 (0.061)		RA	0.198 (0.047)	0.248 (0.047)	0.245 (0.048)	0.293 (0.049)
	LM	0.686 (0.064)	0.559 (0.065)	0.658 (0.061)	0.539 (0.062)		LM	0.307 (0.058)	0.363 (0.059)	0.317 (0.063)	0.312 (0.064)
	RM	0.684 (0.068)	0.568 (0.069)	0.644 (0.065)	0.553 (0.066)		RM	0.286 (0.051)	0.304 (0.051)	0.256 (0.055)	0.309 (0.056)
	CE	0.935 (0.074)	0.772 (0.075)	0.872 (0.069)	0.766 (0.070)		CE	0.258 (0.044)	0.270 (0.045)	0.232 (0.046)	0.274 (0.047)
	LP	1.070 (0.086)	0.853 (0.087)	1.001 (0.082)	0.832 (0.083)		LP	0.214 (0.036)	0.213 (0.036)	0.225 (0.037)	0.204 (0.038)
	RP	1.073 (0.091)	0.888 (0.092)	0.993 (0.086)	0.882 (0.087)		RP	0.214 (0.039)	0.225 (0.040)	0.189 (0.044)	0.240 (0.044)

LA – left anterior; RA – right anterior; LM – left middle; RM – right middle; CE – central; LP – left posterior; RP – right posterior. Items in bold are significant based on p-values.
