# Supplementary material for: Electrophysiological correlates of focused attention on low- and high-distressed tinnitus
Source: PLoS One. 2020 Aug 5;15(8):e0236521. doi: 10.1371/journal.pone.0236521 (PMC7406215; doi:10.1371/journal.pone.0236521)
Supplement: S6 Table — Items in bold are significant based on p-values. (PDF) [file pone.0236521.s006.pdf]

S6 Table. Post-hoc pairwise t-tests results for the contrast: high tinnitus-related distress (HD) versus low tinnitus-related distress (LD) group, calculated in each cluster separately for the body focus condition (BFC) and tinnitus focus condition (TFC). Items in bold are significant based on p-values.

BFC													
		M_{Diff}	CI		p-value	FDR-adjusted p -value			M_{Diff}	CI		p-value	FDR-adjusted p -value
			L	U						L	U		
Delta	LA	0.007	-0.070	0.082	0.837	0.876	Low Beta	LA	0.088	-0.023	0.199	0.119	0.394
	RA	-0.014	-0.093	0.065	0.733	0.797		RA	0.087	-0.030	0.204	0.143	0.394
	LM	0.056	-0.013	0.124	0.108	0.394		LM	0.143	0.017	0.268	**0.026***	0.394
	RM	0.004	-0.071	0.077	0.910	0.926		RM	0.125	-0.005	0.255	0.059	0.394
	CE	0.032	-0.036	0.101	0.354	0.551		CE	0.128	-0.007	0.263	0.062	0.394
	LP	0.056	-0.022	0.133	0.157	0.394		LP	0.158	0.003	0.310	**0.045***	0.394
	RP	0.016	-0.069	0.100	0.698	0.790		RP	0.129	-0.030	0.287	0.112	0.394
Theta	LA	0.048	-0.044	0.140	0.305	0.510	Middle Beta	LA	0.091	-0.017	0.198	0.098	0.394
	RA	0.037	-0.055	0.129	0.428	0.580		RA	0.084	-0.028	0.197	0.143	0.394
	LM	0.084	-0.005	0.173	0.066	0.394		LM	0.113	-0.012	0.239	0.077	0.394
	RM	0.037	-0.057	0.132	0.435	0.580		RM	0.099	-0.032	0.230	0.140	0.394
	CE	0.060	-0.034	0.154	0.211	0.445		CE	0.131	0.001	0.259	**0.048***	0.394
	LP	0.095	-0.008	0.197	0.071	0.394		LP	0.155	0.023	0.286	**0.022***	0.394
	RP	0.063	-0.044	0.171	0.248	0.479		RP	0.123	-0.012	0.256	0.075	0.394
Low Alpha	LA	0.112	-0.071	0.294	0.228	0.455	High Beta	LA	0.077	-0.030	0.189	0.166	0.394
	RA	0.100	-0.088	0.288	0.297	0.510		RA	0.046	-0.055	0.150	0.383	0.567
	LM	0.128	-0.068	0.322	0.198	0.426		LM	0.050	-0.069	0.167	0.402	0.567
	RM	0.096	-0.102	0.294	0.346	0.551		RM	0.057	-0.050	0.167	0.302	0.510
	CE	0.122	-0.079	0.322	0.237	0.465		CE	0.115	0.011	0.221	**0.029***	0.394
	LP	0.197	-0.017	0.411	0.071	0.394		LP	0.128	0.019	0.236	**0.021***	0.394
	RP	0.141	-0.082	0.367	0.219	0.455		RP	0.111	-0.001	0.224	0.053	0.394
High Alpha	LA	0.083	-0.088	0.252	0.336	0.545	Gamma	LA	-0.056	-0.204	0.099	0.467	0.595
	RA	0.080	-0.098	0.255	0.376	0.567		RA	-0.051	-0.176	0.081	0.433	0.580
	LM	0.126	-0.053	0.304	0.166	0.394		LM	-0.056	-0.217	0.102	0.487	0.595
	RM	0.116	-0.072	0.302	0.227	0.455		RM	-0.018	-0.158	0.122	0.797	0.850
	CE	0.162	-0.043	0.366	0.120	0.394		CE	-0.012	-0.135	0.111	0.845	0.876
	LP	0.217	-0.023	0.451	0.075	0.394		LP	0.001	-0.097	0.101	0.985	0.985
	RP	0.185	-0.068	0.434	0.151	0.394		RP	-0.012	-0.121	0.097	0.833	0.876

M_{Diff} – factor score mean difference; CI – 95% confidential interval; L – lower bound; U – upper bound; LA – left anterior; RA – right anterior; LM – left middle; RM – right middle; CE – central; LP – left posterior; RP – right posterior; LA – left anterior; RA – right anterior; * $p < 0.05$; ** $p < 0.01$.

S6 Table continuation

		TFC											
		M $_{Diff}$	CI		p-value	FDR-adjusted p -value			M $_{Diff}$	CI		p-value	FDR-adjusted p -value
			L	U						L	U		
Delta	LA	-0.008	-0.092	0.075	0.867	0.891	Low Beta	LA	0.096	0.055	-0.013	0.083	0.394
	RA	-0.028	-0.110	0.052	0.497	0.595		RA	0.080	0.058	-0.035	0.170	0.394
	LM	0.036	-0.031	0.105	0.293	0.510		LM	0.147	0.062	0.024	**0.019***	0.394
	RM	0.018	-0.055	0.089	0.623	0.712		RM	0.101	0.066	-0.029	0.129	0.394
	CE	0.030	-0.038	0.099	0.385	0.567		CE	0.097	0.066	-0.033	0.142	0.394
	LP	0.063	-0.012	0.140	0.101	0.394		LP	0.142	0.076	-0.008	0.064	0.394
	RP	0.047	-0.036	0.130	0.268	0.500		RP	0.109	0.080	-0.048	0.173	0.394
Theta	LA	0.032	-0.062	0.127	0.512	0.603	Middle Beta	LA	0.085	-0.022	0.192	0.121	0.394
	RA	0.018	-0.077	0.114	0.719	0.794		RA	0.086	-0.024	0.197	0.123	0.394
	LM	0.074	-0.015	0.167	0.106	0.394		LM	0.092	-0.035	0.220	0.156	0.394
	RM	0.037	-0.059	0.135	0.450	0.593		RM	0.089	-0.043	0.222	0.189	0.415
	CE	0.052	-0.042	0.149	0.284	0.510		CE	0.107	-0.020	0.232	0.098	0.394
	LP	0.084	-0.016	0.186	0.100	0.394		LP	0.150	0.020	0.278	**0.025***	0.394
	RP	0.061	-0.046	0.170	0.270	0.500		RP	0.116	-0.015	0.245	0.082	0.394
Low Alpha	LA	0.072	-0.116	0.260	0.457	0.595	High Beta	LA	0.048	-0.053	0.151	0.358	0.551
	RA	0.050	-0.142	0.243	0.617	0.712		RA	0.043	-0.057	0.145	0.410	0.567
	LM	0.106	-0.090	0.301	0.291	0.510		LM	0.091	-0.028	0.211	0.135	0.394
	RM	0.073	-0.129	0.276	0.487	0.595		RM	0.018	-0.095	0.134	0.755	0.813
	CE	0.074	-0.127	0.277	0.479	0.595		CE	0.096	-0.001	0.195	0.052	0.394
	LP	0.152	-0.068	0.373	0.179	0.401		LP	0.148	0.045	0.250	**0.005****	0.394
	RP	0.103	-0.130	0.338	0.396	0.567		RP	0.082	-0.027	0.193	0.141	0.394
High Alpha	LA	0.068	-0.089	0.226	0.395	0.567	Gamma	LA	-0.024	-0.161	0.115	0.723	0.794
	RA	0.058	-0.107	0.223	0.493	0.595		RA	-0.047	-0.177	0.088	0.477	0.595
	LM	0.119	-0.049	0.287	0.168	0.394		LM	0.004	-0.167	0.178	0.972	0.981
	RM	0.091	-0.088	0.270	0.318	0.523		RM	-0.053	-0.208	0.100	0.499	0.595
	CE	0.106	-0.083	0.297	0.273	0.500		CE	-0.042	-0.174	0.084	0.526	0.614
	LP	0.169	-0.060	0.394	0.145	0.394		LP	0.021	-0.082	0.125	0.708	0.793
	RP	0.111	-0.126	0.347	0.359	0.551		RP	-0.051	-0.174	0.069	0.407	0.567

M_{Diff} – factor score mean difference; CI – 95% confidential interval; L – lower bound; U – upper bound; LA – left anterior; RA – right anterior; LM – left middle; RM – right middle; CE – central; LP – left posterior; RP – right posterior; LA – left anterior; RA – right anterior; * $p < 0.05$; ** $p < 0.01$.
